# The Practice of Medicine Defined

**Published:** 1907-04

**Authors:** 


### "THE PRACTICE OF MEDICINE DEFINED."

The Supreme Court of New York recently handed down a very important decision, of which the following is part:

To confine the definition of the words "practice medicine" to the mere administration of drugs or the use of surgical instruments would be to eliminate the very cornerstone of successful medical practice, namely, the diagnosis. It would rule out of the profession those great physicians whose work is confined to consultation, the diagnosticians, who leave to others the details of practice. Section 146 of the Public Health Law provides that persons desiring to practice must pass a Regent's examination made up of "suitable questions for thorough examination in anatomy, physiology and hygiene, chemistry, surgery, obstetrics, pathology and diagnosis and therapeutics, including practice and materia medica." Diagnosis would, therefore, seem to be an integral part of both the study and practice of medicine, so recognized by the law as well as common sense. The correct determination of what the trouble is must be the first step for the cure thereof. It is a well-known fact that the disease popularly known as consumpion may, if discovered in time, be arrested, if not entirely eradicated from the system, by open-air treatment in the proper climate, and that in such cases use of drugs has been practically given up. Would the physician, in such a case, who by his skill discovered the incipient disease, advised the open-air treatment and refrained from administering drugs, not be practicing medicine? It may be difficult by a precise definition to draw the line between where nursing ends and the practice of medicine begins, and the court should not attempt, in construing this statue, to lay down in any case a hard and fast rule on the subject, as the courts have never undertaken to mark the limits of the police power of the state or to have precisely defined what constitutes fraud. What the courts have done is to say that given legislation was or was not within the limits of the police power, or that certain actions were or were not fraudulent.

We are of the opinion, from the general current of the authorities throughout the country and from examination of the history and growth of our own public health statutes, that we should not apply the rule as claimed to have been laid down in Smith vs. Lane. When we find, as in this case, a defendant holding himself out by sign and card as a doctor, with office hours, who talks of his patients and gives treatments, who makes a diagnosis and prescribes diet and conduct and remedies, simple though they be, and who asserts the power to cure all diseases that any physician can cure without drugs and also diseases that they can not cure with drugs, and who takes payment for a consultation wherein there was an examination and determination of the trouble, that is, a diagnosis, as well as payment for subsequent treatment, even if no drugs are administered, we must hold that he comes within the purview of the statute prohibiting the practice of medicine without being lawfully authorized and registered.

